# Dynamics-Adapted Radiotherapy Dose (DARD) for Head and Neck Cancer Radiotherapy Dose Personalization

**DOI:** 10.3390/jpm11111124

**Published:** 2021-11-01

**Authors:** Mohammad U. Zahid, Abdallah S. R. Mohamed, Jimmy J. Caudell, Louis B. Harrison, Clifton D. Fuller, Eduardo G. Moros, Heiko Enderling

**Affiliations:** 1 Department of Integrated Mathematical Oncology, H. Lee Moffitt Cancer Center & Research Institute, Tampa, FL 33612, USA; mohammad.zahid@moffitt.org; 2 Department of Radiation Oncology, The University of Texas MD Anderson Cancer Center, Houston, TX 77030, USA; ASMohamed@mdanderson.org (A.S.R.M.); cdfuller@mdanderson.org (C.D.F.); 3 Department of Radiation Oncology, H. Lee Moffitt Cancer Center & Research Institute, Tampa, FL 33612, USA; Jimmy.Caudell@moffitt.org (J.J.C.); Louis.Harrison@moffitt.org (L.B.H.); eduardo.moros@moffitt.org (E.G.M.)

**Keywords:** radiotherapy, dose personalization, head and neck cancer, mathematical modeling

## Abstract

Standard of care radiotherapy (RT) doses have been developed as a one-size-fits all approach designed to maximize tumor control rates across a population. Although this has led to high control rates for head and neck cancer with 66–70 Gy, this is done without considering patient heterogeneity. We present a framework to estimate a personalized RT dose for individual patients, based on pre- and early on-treatment tumor volume dynamics—a dynamics-adapted radiotherapy dose (*D*_DARD_). We also present the results of an in silico trial of this dose personalization using retrospective data from a combined cohort of *n* = 39 head and neck cancer patients from the Moffitt and MD Anderson Cancer Centers that received 66–70 Gy RT in 2–2.12 Gy weekday fractions. This trial was repeated constraining *D*_DARD_ between (54, 82) Gy to test more moderate dose adjustment. *D*_DARD_ was estimated to range from 8 to 186 Gy, and our in silico trial estimated that 77% of patients treated with standard of care were overdosed by an average dose of 39 Gy, and 23% underdosed by an average dose of 32 Gy. The in silico trial with constrained dose adjustment estimated that locoregional control could be improved by >10%. We demonstrated the feasibility of using early treatment tumor volume dynamics to inform dose personalization and stratification for dose escalation and de-escalation. These results demonstrate the potential to both de-escalate most patients, while still improving population-level control rates.

## 1. Introduction

Head and neck cancers (HNC) are among the ten most common cancer types worldwide, with an increasing incidence in certain virally driven subtypes [1,2]. Established risk factors for HNC include tobacco use, alcohol consumption and infection by the human papillomavirus (HPV). Standard of care treatment options include definitive RT, with or without systemic therapy, or initial surgical resection followed by adjuvant RT, with or without systemic therapy, as needed and based on pathological risk features [3]. For HNC, the standard RT protocol delivers a total of 66–70 Gy in 30–35 weekday fractions of 1.8–2 Gy each. Treatment with definitive RT, with or without systemic therapies, has a high cure rate of 50–95%, but this comes with potential RT-associated late toxicities such as osteoradionecrosis, dysgeusia, neuropathies, tooth decay, dysphagia, or feeding tube dependency [4]. One obvious shortcoming of current clinical practice is that RT is planned without regard to any of the patient-specific factors that may influence outcome. With an increasing understanding of inter-patient heterogeneity, RT should be tailored to individual patients [5]. Current efforts to personalize RT mainly adapt the target volume based on response; however, there have been no trials attempting to individualize radiation dose.

There is a broad interest in identifying which HNC patients, especially those with HPV+ oropharyngeal cancers, as they may be candidates for receiving less than the standard 66–70 Gy RT dose, in order to minimize radiation-induced toxicities. This is evidenced by the numerous trials, meta-analyses, and opinion pieces on the topic [6,7,8]. However, uniform dose de-escalation runs the risk of lower population-level tumor control rates. Personalization of radiation dose may hold the key to improve overall outcomes, and any step to deliver individualized radiation must be backed up by solid scientific evidence.

There have been attempts to identify candidates for dose de-escalation via hypoxia imaging [9] and analyses of combination of clinical variables such as HPV status and smoking history. Additionally, there is a growing body of work in identifying genomic markers of RT sensitivity, which have been suggested as a means to personalize radiation dose for individual patients [10,11]. All of these approaches are limited by the fact that they attempt to classify or stratify based on the information from a single time point, and that they are generally informed by unifactorial metrics. However, response to RT is a complex multifactorial phenomenon that needs to be treated as an emergent property arising from the dynamic interplay of all these factors. It has recently been postulated that the future of personalized RT, including RT for HNC, will need to integrate and synergize clinical radiation oncology with the expertise of mathematics [12,13,14].

Modeling tumor volume dynamics can reveal an emergent description of “tumor radiosensitivity”. The recent development of the Proliferation Saturation Index, *PSI*, has demonstrated the utility of tumor growth and response dynamics to provide actionable insights into radiation responses [15,16,17]. We have recently extended this concept, and presented that measurements of tumor volume changes during the early weeks of RT can be used in a forecasting framework to make predictions of patient outcomes [18]. Here, we present a framework for using tumor volume dynamics to adapt RT dose for individual patients and the results of an in silico trial of personalized dose adaptation for head and neck cancer.

## 2. Materials and Methods

### 2.1. Patient Data

**Figure 1:**
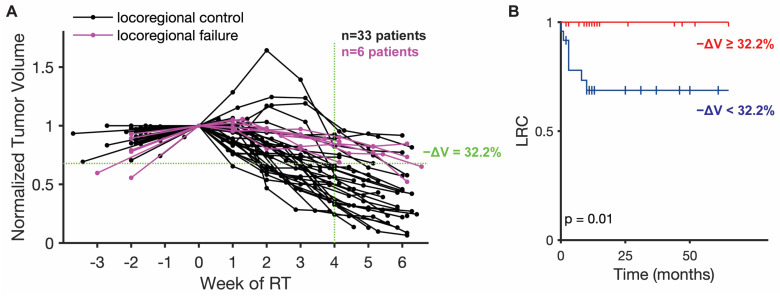
**Patient tumor volume trajectories and correlation with locoregional control (LRC).** (**A**) Longitudinal tumor volume trajectories for all 39 patients normalized by initial patient tumor volume at start of RT with one measurement before the start of RT and weekly measurements during the course of treatment. Patients with eventual locoregional failure are highlighted in purple. The indicated median volume reduction (−ΔV = 32.2%) at week 4 of RT perfectly separates the patients with locoregional control (LRC) and locoregional failure. (**B**) Kaplan–Meier survival plot for locoregional control (LRC) separated by percent tumor volume reduction (−ΔV = 32.2%) at 4 weeks of RT.

Longitudinal tumor volume data were collected for a cohort of 17 head and neck cancer patients from the Moffitt Cancer Center (MCC) treated with 66–70 Gy RT in 2 Gy weekday fractions, and for a cohort of 22 patients from the MD Anderson Cancer Center (MDACC) with 66–70 Gy RT in 2 or 2.12 Gy weekday fractions or with accelerated fractionation with or without chemotherapy (Figure 1A). Tumor volume measurements were derived from cone beam computed tomography (CBCT) scans for the MCC cohort and from CT scan from a CT-on-Rails combining a GE Smart Gantry CT scanner (General Electric, Boston, MA, USA) and a Varian 2100EX linear accelerator (Varian Medical Systems, Palo Alto, CA, USA). For both cohorts, scans were collected at the time of RT planning, just before the first RT dose, and weekly scans during the course of treatment. All CT images were loaded into Mirada imaging software (Mirada Medical, Denver, CO, USA) and primary tumor and/or involved lymph nodes were contoured by a single physician (JJC).

Locoregional control (LRC), defined as time without recurrence or cancer in the treated fields, was abstracted as an outcome measure and determined by biopsy confirmation or radiological imaging with a median follow-up time of 12 months (mean of 20 months). The mid-treatment tumor volume reduction (after 4 weeks of RT) can be thresholded to separate the combined MCC and MDACC cohort into two risk strata for locoregional failure (Figure 1B). The high-risk stratum is defined as patients with mid-treatment tumor volume reduction less than the threshold value of 32.2% reduction. The patients in this stratum have a probability of locoregional failure of approximately 40%. The low-risk stratum is defined as patients with mid-treatment tumor volume reduction greater than the threshold value; these patients all have LRC. The survival data for these two strata were compared using the log rank statistical test to test the null hypothesis that there is no difference between the survival curves, and this test yielded a *p*-value of 0.01. This stratification is in line with the prognostic value of mid-treatment nodal response observed by other groups [19]. The definition of these strata enables the relation of tumor volume changes and estimated personalized RT dose with patient outcomes.

### 2.2. Mathematical Model

Tumor growth was modeled as logistic growth as described by the following equation:

$$\frac{dV}{dt}=\lambda V\left(1-\frac{V}{K}\right)$$

where V(t) is tumor volume [cc], λ is the volumetric growth rate [day^−1^], and K(t) is the tumor carrying capacity [cc], which is defined as the maximum tumor size that the local tissue can support at time *t*.

The effect of a single fraction of radiation on the tumor is modeled as an instantaneous reduction in the tumor carrying capacity [18], as described by the following equation,

$$K_{+}=K_{-}\left(1-\delta \right)$$

where $K_{-}$ and $K_{+}$ are, respectively, the carrying capacities immediately before and after each radiation fraction. The δ parameter describes the carrying capacity reduction fraction, which varies between 0 and 1, where 0 means there is no reduction in the carrying capacity and 1 means there is a 100% reduction in the carrying capacity.

### 2.3. Model Calibration and Fitting

The mathematical model was calibrated using the longitudinal tumor volume data from the 39 patients from MCC and MDACC. Tumor carrying capacity prior to radiation, $K_{0}$, was calculated using 2 pre-treatment tumor volume measurements with the following equation

$$K_{0}=\frac{V_{0}\cdot V_{plan}\left(e^{\lambda \Delta t}-1\right)}{V_{plan}\cdot e^{\lambda \Delta t}-V_{0}}$$

where $V_{0}$ is the tumor volume right before the start of RT treatment, $V_{plan}$ is the tumor volume calculated from the treatment planning computer tomography (CT; commonly known as a CT-sim for simulation) scan (usually a few days to weeks before start of RT), and Δt is the time between the treatment planning CT and the start of RT.

The initial proliferative state of the tumor is characterized by the proliferation saturation index (*PSI*), which has been defined as the ratio of the initial tumor volume and tumor carrying capacity prior to the first RT dose:

$$PSI\;\equiv \frac{V_{0}}{K_{0}}.$$

*PSI* represents the proportion of non-proliferative cells in the tumor volume: if *PSI* = 0, then the entire volume consists of proliferative cells yielding exponential tumor growth, and if *PSI* = 1 the entire tumor volume is non-proliferative in a state of population-level dormancy.

Model fitting was performed using patient-specific $K_{0}$ and a fixed value of λ = 0.13 day^−1^ for all patients, which was optimized in the original presentation of this model [18]. Patient-specific values for δ were then determined using the particle swarm optimization toolbox in MATLAB with δ being bound between 0 and 1, as per the definition of the parameter. Model fit to data was analyzed using normalized root mean square error, <nRMSE>.

### 2.4. Dose Personalization Framework

**Figure 2:**
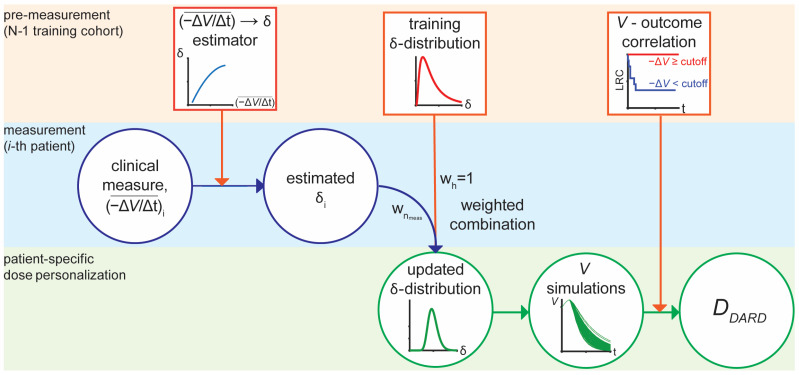
**RT Dose personalization framework for *D*_DARD_.** The framework is divided into three phases: Pre-measurement based on the historical cohort, measurements for the i-th patient, and patient-specific dose personalization. The squares represent information learned from the training cohort; circles represent information measured or calculated for an individual patient.

The mathematical model was used to determine the dynamics-adjusted radiation therapy dose, *D*_DARD_, which is the minimum cumulative RT dose predicted for LRC. This was done using a framework that was adapted from the original implementation that was used to forecast patient outcomes with high specificity and sensitivity using a few weeks of on-treatment tumor volume measurements [18]. The framework learns 3 inputs from a training cohort: (1) a function to estimate *δ* from average weekly tumor volume decrease, $\bar{-\Delta V/\Delta t}$, (2) a prior distribution for *δ*, and (3) a volume reduction cutoff correlated with the patient outcome of interest, in this case LRC (Figure 2).

The ($\bar{-\Delta V/\Delta t})\to \delta$ estimator takes the form of the following quadratic relation

$$\delta =\beta_{1}\;{\left(\;\bar{-\frac{\Delta V}{\Delta t}}\;\right)}^{2}+\beta_{2}\;\left(\;\bar{-\frac{\Delta V}{\Delta t}}\;\right)+\beta_{3}$$

where $\beta_{1}$, $\beta_{2}$, and $\beta_{3}$ coefficients are learned through least-squares regression. The prior distribution for δ is assumed to be distributed according to a log-normal distribution and fit to the fitted δ values from the training cohort using the MATLAB Distribution Fitting Application. The volume reduction cutoffs were derived by testing 100 possible cutoff values, spanning the entire range of possible cutoffs and selecting the cutoffs that minimized the log rank *p*-values in order to maximize the significance of curve separation of the LRC Kaplan–Meier survival curves. This yields an optimal volume reduction threshold at week 4 of RT-associated with LRC in the training cohort with a range of (22.9–31.3)% volume reduction.

The mean weekly tumor volume reduction for the current patient, ${\left(\bar{-\frac{\Delta V}{\Delta t}}\right)}_{i}$ is input to the learned ($\bar{-\Delta V/\Delta t})\to \delta$ estimator to generate a patient-specific estimate of δ. This estimate is combined with the prior δ-distribution to generate a patient-specific posterior δ-distribution:

$$Lognormal\;\sim \left(\mu_{i}=\frac{w_{h}\cdot \mu_{h}+w_{n_{meas}}\cdot ln\;\left(\delta_{i}\right)\cdot n_{meas}}{w_{n}{}_{meas}\cdot n_{meas}+1},\;\sigma_{i}=\frac{\sigma_{h}}{n_{meas}+1}\right),$$

where $\mu_{i}$ and $\sigma_{i}$ are the updated parameters for the patient-specific posterior δ-distribution; $\mu_{h}$ and $\sigma_{h}$ are the parameters for the prior δ-distribution; $w_{n_{meas}}\in \left(0,10\right)$ is the weight given to the patient’s clinical measures relative to a weight of $w_{h}=$1 given to the prior δ-distribution for the *n*-th clinical measurement; and $n_{meas}$ is the number of measurements being considered in a given prediction (here $n_{meas}$ = 4, as 4 on-treatment measurements are used to estimate $\delta_{i}$). This particular design allows the distribution to shift towards $\delta_{i}$ and to narrow as the number of measurements increases.

Tumor volume trajectories are generated by sampling from the updated δ-distribution 100 times to create a forecast of the tumor’s response to an additional 16 weeks of RT. While this is far longer than typical clinical RT treatment courses, but this is done to calculate an initial estimate of *D*_DARD_. *D*_DARD_ is determined by measuring cumulative dose that includes the RT fraction such that all of the forecasted tumor volume trajectories have a tumor volume reduction below the cutoff association with complete LRC.

It should be noted, that if the estimated value of $\delta_{i}$ < 0, due to the average weekly tumor volume decrease being much smaller than what was seen in the training cohort, then δ is sampled directly from the prior distribution. Additional technical details about the framework, such as details of how weights were optimized, can be found in the original presentation of the forecasting framework [18].

### 2.5. Dose Personalization In Silico Trial Design

**Figure 3:**
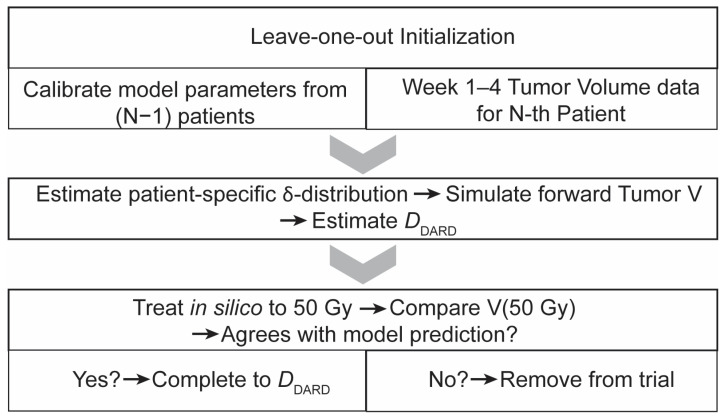
**Flowchart description of in silico trial sequence.** The trial has three major phases: (1) Leave-one-out initialization, where model parameters are calibrated from the training cohort and combined with tumor volume data from before the start of RT to week 4 of RT, (2) Personalized dose estimation, and (3) Safety check for model agreement with measured tumor volume after in silico treatment up to 50 Gy.

To test the framework for RT dose personalization, we designed an in silico trial that mimics the potential implementation of mathematics-guided dose personalization in a single-arm study (Figure 3).

Since locoregional failures were rare (<16%) in the dataset, this in silico trial was run in a leave-one-out approach, where the framework was trained on N-1 patients and then used to find a personalized dose for the N-th patient. This process was then repeated similarly for the remaining patients. This type of analysis is classified as a type 1b analysis in the TRIPOD recommendations for predictive models, which is considered appropriate for model development and internal validation in the context of limited data [20].

Each patient is simulated to receive 4 weeks of the clinically applied RT dosing schema (1.8–2 Gy daily weekday fractions). At the start of week 5 of RT, tumor volume data from weeks 1–4 of RT are input to the dose personalization framework in order to calculate an initial estimate of *D*_DARD_ for the virtual patient. We then further simulated RT to a cumulative dose of 50 Gy, after which the clinically observed tumor volume measurement was compared to the model prediction made at the beginning of week 5 of RT. If the in silico tumor volume trajectories were on the same side of the LRC threshold as the measured tumor volume after 50 Gy of RT, then the in silico treatment was completed to *D*_DARD_. Otherwise, in silico treatment reverted to standard of care, and the virtual patient received the same dose that the original patient received.

## 3. Results

### 3.1. Model Fitting

**Figure 4:**
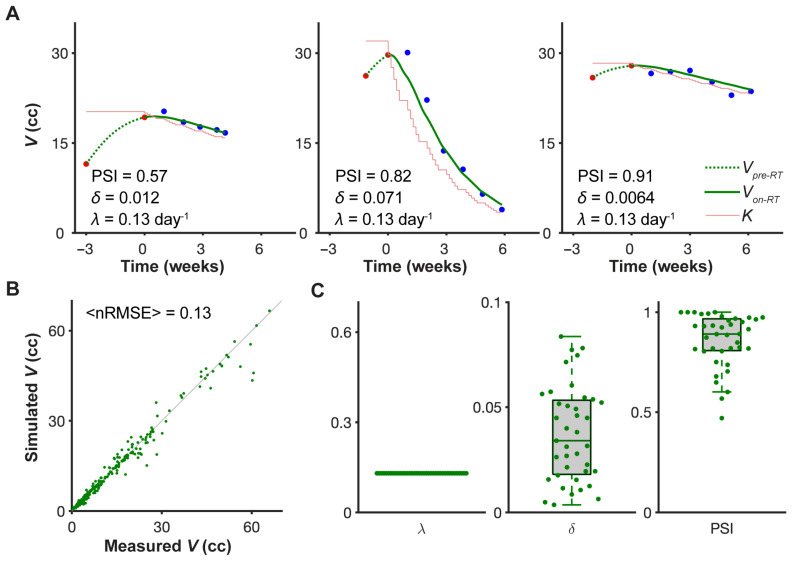
**Model fit to longitudinal tumor volume data.** (**A**) Representative model fits for three patients arranged in order of increasing PSI values. Red dots are measured pre-treatment tumor volumes; black dots on-treatment tumor volumes; the dashed green curves are the calculated pre-treatment tumor growth trajectory; the solid green curves are the fitted on-treatment tumor volume trajectories; and the thin red line indicates the calculated value of the tumor carrying capacity both before and during treatment. (**B**) Correlation of measured tumor volumes and fitted tumor volumes for all 39 patients with indicated average normalized root mean square error (<nRMSE>). Green dots indicate individual weekly tumor volumes. (**C**) Parameter distributions for all 39 patients. Volumetric tumor growth rate, *λ* = 0.13 day^−1^, was fixed for all patients.

The mathematical model fits the different on-treatment tumor volume dynamics for the 39 head and neck cancer patients from MCC and MDACC with high accuracy (<nRMSE> = 0.13), using only two patient-specific parameters (Figure 4A,B). The growth rate λ was kept fixed at 0.13 day^−1^ across all patients, and although the optimization algorithm search for δ over the whole range of (0,1), the fitted values of δ were all <0.1 (Figure 4C). The model fitting results were robust across a range of pre-treatment volume dynamics, as captured by the range of *PSI* values (0.47,1). Notably, we did not account for whether or not the patients received chemotherapy, so the effect of chemotherapy is also captured in the patient-specific fit of the δ parameter.

### 3.2. Personalized Dynamics-Adapted Radiation Therapy Dose (D_DARD_)

**Figure 5:**
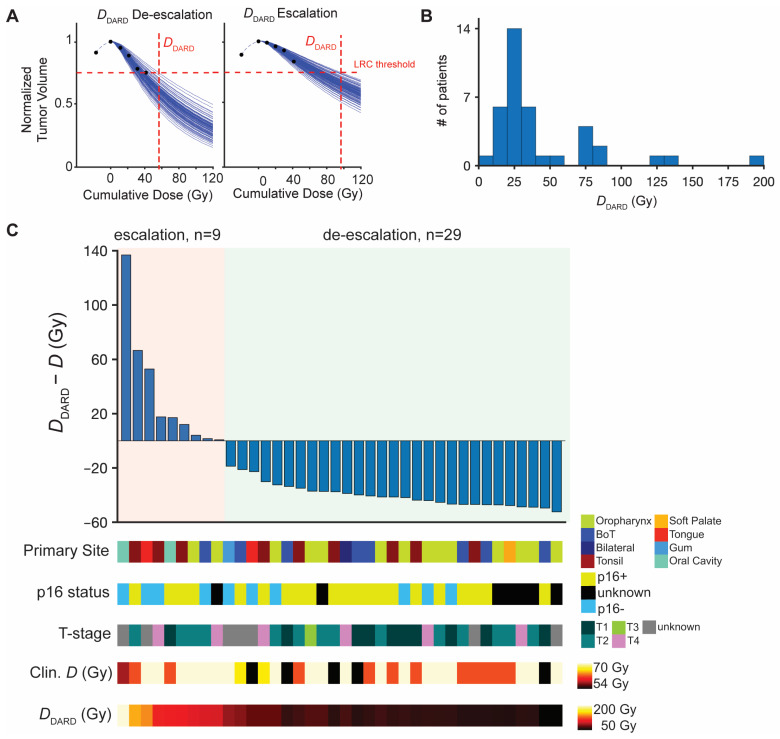
**Dose personalization and trial results using *D*_DARD_.** (**A**). Example calculations of finding minimum RT dose for locoregional control, *D*_DARD_, for two patients using 4 tumor volume measurements (1 before start of RT and 4 from weeks 1–4 of RT). Black dots are normalized tumor volume measurements; blue curves the 100 projected tumor volume forecasts; horizontal dashed line the volume reduction threshold associated with LRC; and the vertical dashed line *D*_DARD_, the minimum dose where all 100 trajectories are below the LRC threshold. (**B**). Histogram of calculated *D*_DARD_ values for all 39 patients. (**C**). Waterfall plot of difference between *D*_DARD_ and the actual dose received in the clinic, where ΔD > 0 indicates dose escalation and ΔD < 0 indicates dose de-escalation for the 38 patients on the trial. Individual patient characteristics of interest (primary tumor site, p16 status, T-stage, cumulative dose received in the clinic, and *D*_DARD_) are indicated for each patient below the waterfall plot.

During the second phase of the in silico trial, we calculate the minimal required dose, *D*_DARD_, to achieve a tumor volume reduction below the trained cutoff for locoregional control. Compared to the clinically delivered total dose, *D*, *D*_DARD_ indicates candidates for dose escalation if *D*_DARD_ > *D*, or de-escalation if *D*_DARD_ < *D* (Figure 5A). *D*_DARD_ ranges from 8–186 Gy (Figure 5B) and suggests that 77% (*n* = 30) of patients treated with standard of care were overdosed by an average dose of 39 Gy, and 23% (*n* = 9) underdosed by an average dose of 32 Gy (Table 1). One patient was predicted to not achieve the necessary tumor volume reduction for LRC within 20 weeks of in silico RT to a cumulative total dose of 200 Gy.

During the final phase of the in silico trial, only one patient was removed from the trial due to disagreement between the model prediction and measured tumor volume at 50 Gy. The relative dose changes (*D*_DARD_—*D*) for the remaining 38 patients are summarized in Figure 5C. Although the small size of the cohort limits statistical comparisons with clinical characteristics, we visualized the distribution of the primary tumor site, T-stage, p16 viral status, and the originally delivered RT dose for the predicted escalation and de-escalation cohorts. Interestingly, there were patients with T4 tumors in both the escalation and de-escalation subgroups. The 9 patients predicted for dose escalation had a variety of disease sites (tonsil [3], oral cavity [2], tongue [1], base of tongue [1], oropharynx [2]). Of interest, 4 predicted escalation patients (44%) were p16-, 4 were p16+, and 1 unknown. Similarly, of the patients with de-escalation *D*_DARD_, 18 were p16+ (60%) and 6 were p16- (20%; 6 with unknown p16 status), suggesting that HPV status alone may not be a clear indicator for HNC dose personalization.

### 3.3. $D_{\mathrm{DARD}}^{*}:$Dose Personalization within Restricted Dose Range

**Figure 6:**
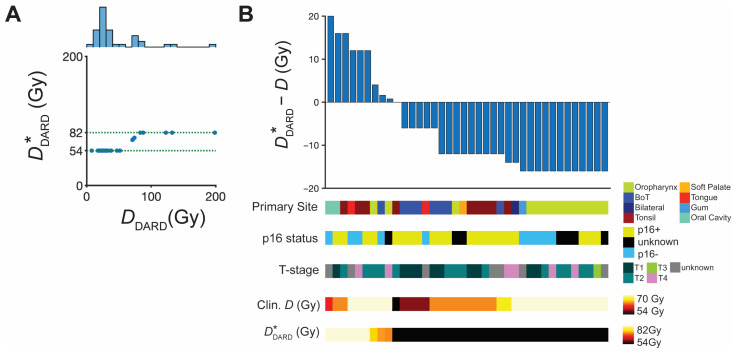
**Dose adjustment to** $D_{\mathrm{DARD}}^{*}$and subsequent in silico trial results. (**A**) Scatter plot of $D_{\mathrm{DARD}}^{*}$ and *D*_DARD_ limited to the moderate escalation/de-escalation range of (54,82) Gy for 38 patients. A histogram of the *D*_DARD_ is projected above the scatterplot. (**B**) Waterfall plot of difference between $D_{\mathrm{DARD}}^{*}$ and the actual dose received in the clinic, where ΔD > 0 indicates dose escalation and ΔD < 0 indicates dose de-escalation for the 38 patients that remained on the trial. Individual patient characteristics of interest (primary tumor site, p16 status, T-stage, cumulative dose received in the clinic, and $D_{\mathrm{DARD}}^{*}$) are indicated for each patient below the waterfall plot.

*D*_DARD_ suggests a widespread of personalized radiation doses. As most of the *D*_DARD_ values are far outside the current standard of care prescription dose of 66–70 Gy for HNC, which may be unrealistic to test in initial clinical trials, we tested a restricted personalized dose range of (54,82) Gy based on upper and lower limits tested in previous clinical trials of locally advanced HNC and HPV-associated oropharyngeal cancer [21,22,23]. Thus, we mapped *D*_DARD_ < 54 Gy to $D_{\mathrm{DARD}}^{*}$ = 54 Gy and *D*_DARD_ > 82 Gy to $D_{\mathrm{DARD}}^{*}$ = 82 Gy (Figure 6A) and repeated the in silico trial with $D_{\mathrm{DARD}}^{*}$. The results of this trial are summarized in Figure 6B.

The majority of patients remained candidates for dose de-escalation (*n* = 29; 74%) with an average de-escalation of 12.5 Gy per patient. Dose escalation patients (*n* = 9; 23%) would be treated with an average dose escalation of 10.7 Gy per patient. Additionally, the LRC control rate is predicted to improve by a clinically significant >10% (*n* = 4 patients; Table 1), compared to the standard dosing the patient received in the clinic.

## 4. Discussion

We developed a mathematical modeling framework to introduce the concept of a dynamics-adapted radiation therapy dose for individual HNC patients. The results of the in silico trials with both $D_{\mathrm{DARD}}$ and the constrained $D_{\mathrm{DARD}}^{*}$ show the feasibility of using early treatment tumor volume dynamics to inform dose personalization and stratification for dose escalation and de-escalation. These results demonstrate the potential to both de-escalate the majority of patients, while still improving population-level control rates. Additionally, these in silico results provide evidence to bolster the hypothesis that dose personalization, rather than uniform de-escalation or escalation across entire populations/subpopulations, provides a promising way forward to both improve patient quality of life while maintaining the progress that has been achieved with high cure rates with definitive RT in HNC. Additionally, the lack of correlation of primary site, T-stage, or p16 status with either the escalation or de-escalation subgroups suggests that uniform dose escalation or de-escalation of more and more precisely defined subpopulations would be unsuccessful. Although this particular conclusion must be taken cautiously, given the limited size of the cohort.

The dose personalization methodology presented herein could potentially be applied in any treatment setting where fractionated RT is used with a potential for dynamics-informed dose adaptation. The advantage of applying this methodology to fractionated delivery of RT is that this context allows for enough time to observe, calibrate the model, make forecasts, and then adjust the treatment course.

We imagine that for a real implementation of the dose personalization methodology in the clinic, it will be best to make combined predictions using other data types, such as genomic signatures of tumor cell radiosensitivity, hypoxia imaging, HPV status, smoking history, radiomics, etc. However, one of the major advantages of this approach is that it is entirely built on tumor volume data that can be acquired from routine CT/CBCT images.

Given the limited nature of the dataset, both in terms of total numbers and low number of failures, these results need external and prospective validation using data from larger independent cohorts. However, the results of this study provide a conceptual framework for a dose personalization trial that would allow us to take the first steps towards personalized RT dosing for HNC. While $D_{\mathrm{DARD}}^{*}$ with dose personalization within the dose range of standard of care for HNC may only yield an average de-escalation of 10.7 Gy per patient, this may provide a significant improvement toward limiting RT-associated co-morbidities. For instance, it has been observed that in oropharyngeal cancer every additional 10 Gy of RT increases the probability of dysphagia by 19% [24], so even moderate dose de-escalation may lead to significant improvements in patient quality of life.

Possible future studies could explore further in silico testing of RT personalization. This could include testing alternative fractionation schemes with the potential for personalized or dynamic fractionation (i.e., varying fraction size through the course of treatment). Additionally, if the inclusion of systemic therapies can be encoded in the carrying capacity reduction parameter, then it may be possible to predict which patients may benefit from the inclusion of such therapies at different stages in their treatment course. However, all of these investigations would be limited by appropriate data for model calibration and validation, to ensure confidence in the model predictions and recommendations [25].

Finally, while the focus of the presented work is on HNC, it is conceivable that the DARD framework is translatable to other cancer types. Model training, calibration, and predictive power validation would need to be done on each cancer site before in silico and ultimately prospective clinical validation. We recognize that these models would need to be validated on prospective clinical trials, but we feel that results of these model analyses serve to establish the biological rationale to motivate and guide the design of such trials.

## 5. Patents

HE and MUZ are inventors on a provisional patent application entitled “Personalized Radiation Therapy” (63/010,327).

## Figures and Tables

**Table 1:** Summary of dose personalization and estimated effect on LRC.

	Clinical D	$D_{\mathrm{DARD}}$	$D_{\mathrm{DARD}}^{*}$
Mean Escalation (Gy)	0	38.9	12.4
Mean De-Escalation (Gy)	0	32.0	10.7
LRC Rate	84.6%	100%	94.9% ^1^

^1^ Estimated based on number of escalated patients for whom $D_{\mathrm{DARD}}^{*}=D_{\mathrm{DARD}}$

## Data Availability

Code and materials used for this paper are available upon reasonable request.
